# Antioxidants inhibit advanced glycosylation end-product-induced apoptosis by downregulation of miR-223 in human adipose tissue-derived stem cells

**DOI:** 10.1038/srep23021

**Published:** 2016-03-11

**Authors:** Zhe Wang, Hongqiu Li, Ran Guo, Qiushi Wang, Dianbao Zhang

**Affiliations:** 1 Department of Blood Transfusion, Shengjing Hospital of China Medical University, Shenyang 110004, China; 2 Department of Orthopedics, Central Hospital of Shenyang Medical College, Shenyang 110024, China; 3 Department of Orthopaedics, Shengjing Hospital of China Medical University, Shenyang 110004, China; 4 Department of Stem Cells and Regenerative Medicine, Key Laboratory of Cell Biology, Ministry of Public Health, China medical University, Shenyang 110001, China

## Abstract

Advanced glycosylation end products (AGEs) are endogenous inflammatory mediators that induce apoptosis of mesenchymal stem cells. A potential mechanism includes increased generation of reactive oxygen species (ROS). MicroRNA-223 (miR-223) is implicated in the regulation of cell growth and apoptosis in several cell types. Here, we tested the hypothesis that antioxidants N-acetylcysteine (NAC) and ascorbic acid 2-phosphate (AAP) inhibit AGE-induced apoptosis via a microRNA-dependent mechanism in human adipose tissue-derived stem cells (ADSCs). Results showed that AGE-HSA enhanced apoptosis and caspase-3 activity in ADSCs. AGE-HSA also increased ROS generation and upregulated the expression of miR-223. Interestingly, reductions in ROS generation and apoptosis, and upregulation of miR-223 were found in ADSCs treated with antioxidants NAC and AAP. Furthermore, miR-223 mimics blocked antioxidant inhibition of AGE-induced apoptosis and ROS generation. Knockdown of miR-223 amplified the protective effects of antioxidants on apoptosis induced by AGE-HSA. miR-223 acted by targeting fibroblast growth factor receptor 2. These results indicate that NAC and AAP suppress AGE-HSA-induced apoptosis of ADSCs, possibly through downregulation of miR-223.

Human adipose tissue-derived stem cells (ADSCs) are multipotent stromal cells in adipose tissue. Emerging evidence has shown the beneficial effects of ADSC administration to treat various diseases^1^. Furthermore, ADSCs have been found to promote wound healing^2^. Diabetes is associated with an impaired ability to heal wounds. Accordingly, promotion of wound healing by stem cell therapy, which is observed in non-diabetic conditions, is significantly attenuated in diabetic patients^3^. Although autologous ADSC administration has been reported to improve healing in diabetic skin repair, impairment of resident and recruited stem cell functions strongly contributes to delays in wound healing under diabetic conditions^4^,^5^,^6^. However, approaches have not been developed to improve ADSC functions in diabetic individuals.

Previous studies have implicated advanced glycosylation end-products (AGEs) in impaired diabetic wound healing^7^. AGEs are a group of heterogeneous compounds formed by the Maillard reaction, which starts from stiff bases and the Amadori product, α 1-amino-1-deoxyketose, produced by the reaction of the carbonyl group of a reducing sugar. The Maillard reaction involves proteins via non-enzymatic glycation, lipids, and nucleic acids by reducing sugars and aldehydes. During Amadori reorganization, these highly reactive intermediate carbonyl groups, recognized as α-dicarbonyls or oxoaldehydes, products of which induce 3-deoxyglucosone and methylglyoxal, tend to accumulate^8^. Recent studies indicate that AGE modification of proteins may lead to alterations of normal functions by inducing cross-linking of extracellular matrices. Intracellular formation of AGEs can also cause generalized cellular dysfunction. Furthermore, AGEs can mediate their effects via specific receptors, such as the receptor for AGE (RAGE), thus activating diverse signal transduction cascades and downstream pathways, including generation of reactive oxygen species (ROS). Oxidative stress occurs as a result of the imbalance between ROS production and antioxidant defenses. Sources of ROS include mitochondria, auto-oxidation of glucose, and enzymatic pathways, which include nicotinamide adenine dinucleotide phosphate reduced (NADPH) oxidase^9^,^10^. Apoptosis is a potential mechanism through which AGEs exert their effects on cellular dysfunction^11^,^12^. It has been shown that AGEs induce apoptosis in mesenchymal stem cells (MSCs) and endothelial progenitor cells (EPCs)^13^. Increases in MSC apoptosis contribute to delayed wound healing in diabetic rats^14^. Excessive production of ROS plays an important role in apoptosis^15^. It has been reported that AGEs induce MSC apoptosis through overproduction of intracellular ROS^11^.

L-Ascorbic acid 2-phosphate (AAP) is an oxidation-resistant derivative of ascorbic acid. It has been demonstrated that AAP promotes cell differentiation and DNA synthesis. N-acetyl-L-cysteine (NAC) is a prodrug/precursor of the biological antioxidant glutathione. It is a potent ROS inhibitor and has been widely used to counter the adverse effects of oxidative stress^16^. However, the mechanism by which NAC and AAP protect cells from oxidative stress has not been fully elucidated. Recently, several microRNAs (miRNAs) have been found to interfere with and modulate intracellular apoptosis signaling^17^,^18^,^19^,^20^. In the current study, we employed NAC and AAP as antioxidants to reduce oxidative stress levels and apoptosis in ADSCs exposed to AGEs, and focused on how the protective effects are modulated by miRNAs for a potentially new therapeutic approach.

## Results

### Antioxidants suppress AGE-HSA-induced apoptosis and caspase-3 activity in ADSCs

**Figure 1:**
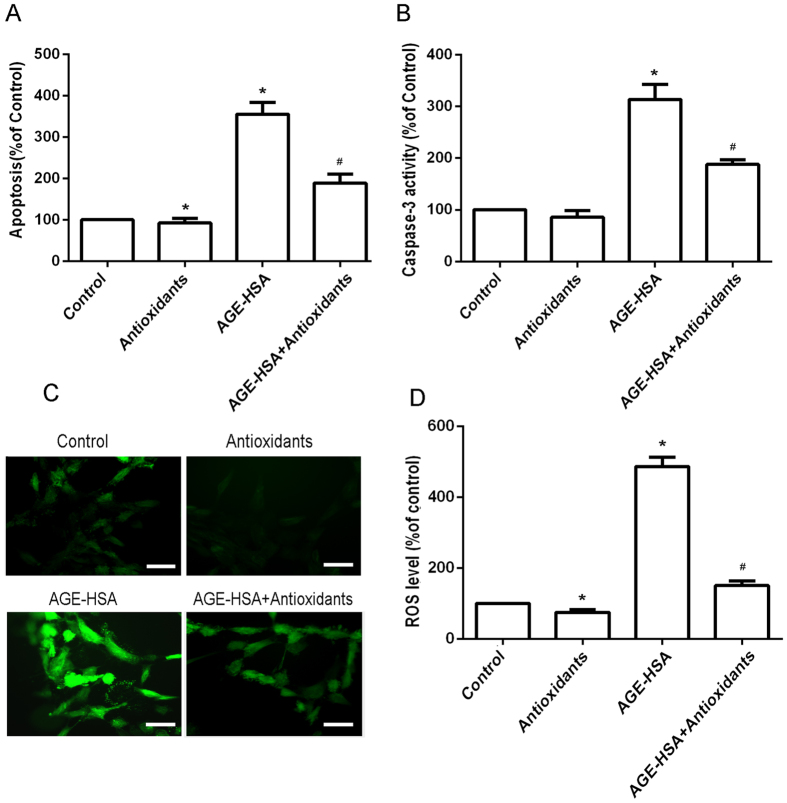
Effects of antioxidants on AGE-HSA-induced apoptosis, caspase-3 activity, and ROS generation in ADSCs. Cells were pretreated with antioxidants followed by treatment with AGE-HSA (300 μg/ml) for 24 h. The level of apoptosis (**A**) and caspase-3 activity (**B**) were measured by ELISA. (**C**) Intracellular ROS generation was visualized under the fluorescence microscope. The scale bars represent 100 μm. (**D**) The level of DCF-sensitive ROS was measured by a flow cytometer. Each value is expressed as the mean ± SD of three independent experiments. *P < 0.05 vs. control (HSA 300 μg/ml). ^#^P < 0.05 vs. AGE-HSA-treated group (300 μg/ml).

Cells were treated with HSA (300 μg/ml) or AGE-HSA (300 μg/ml) for 24 h. As shown in Fig. 1A, the cells treated with AGE-HSA showed an increase in apoptotic cell death compared with control cells. To determine whether antioxidants affect AGE-HSA-induced apoptosis and caspase-3 activity of ADSCs, the cells were pretreated with 3 mM NAC and 0.2 mM AAP for 20 h and then treated with AGE-HSA (300 μg/ml) for 24 h. The levels of apoptosis and caspase-3 activity of ADSCs were then determined by enzyme-linked immunosorbent assays (ELISAs). We found that the antioxidants significantly suppressed AGE-HSA-induced apoptosis (P < 0.05) (Fig. 1A). Caspase-3 is the principal effector caspase through which the mitochondrial and cytosolic pathways induce apoptosis. Therefore, we measured the levels of caspase-3 activity in each group (Fig. 1B). AGE-HSA significantly increased caspase-3 activity in treated cells compared with control cells (P < 0.05). Interestingly, antioxidants significantly suppressed the AGE-HSA-induced caspase-3 activity of ADSCs (P < 0.05).

### Antioxidants suppress oxidative stress levels in ADSCs induced by AGE-HSA

The effects of AGEs on apoptosis of ADSCs are mediated by production of ROS. To determine whether antioxidants affect ROS production, the cells were pretreated with 3 mM NAC and 0.2 mM AAP for 20 h and then treated with AGE-HSA (300 μg/ml) for 24 h. Production of ROS was then determined by DCFH-DA. As shown in Fig. 1C,D, the antioxidants significantly suppressed ROS production in ADSCs induced by AGE-HSA (P < 0.05).

### Antioxidants suppress upregulation of miR-223 in ADSCs induced by AGE-HSA

**Figure 2:**
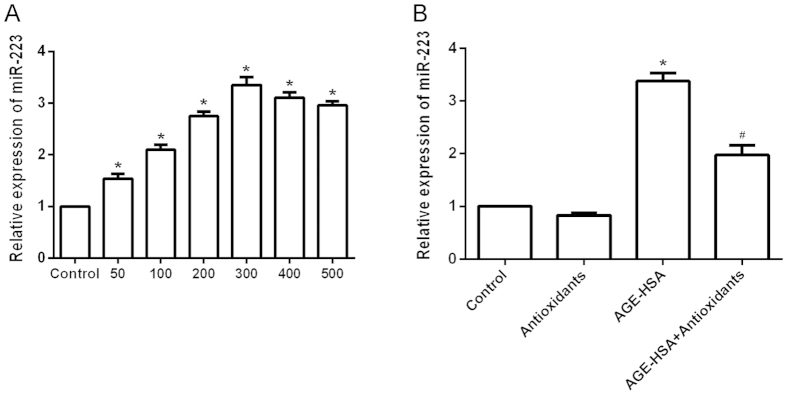
Effects of antioxidants on AGE-HSA-induced upregulation of miR-223 in ADSCs. (**A**) Cells were incubated with HSA (300 μg/ml) or AGE-HSA (50–500 μg/ml) for 24 h. (**B**) Cells were pretreated with antioxidants followed by treatment with AGE-HSA (300 μg/ml) for 24 h. The mRNA expression level of miR-223 was analyzed by RT-PCR. Each value is expressed as the mean ± SD of three independent experiments. *P < 0.05 vs. control (300 μg/ml HSA). ^#^P < 0.05 vs. AGE-HSA-treated group (300 μg/ml).

MiR-223 has been found to interfere with and modulate intracellular apoptosis signaling. To determine whether AGE-HSA affects the expression of miR-223 in ADSCs, the cells were treated with HSA (300 μg/ml) or AGE-HSA (50–500 μg/ml) for 24 h, and then the expression of miR-223 was determined by RT-PCR. As shown in Fig. 2A, cells treated with AGE-HSA showed an increase in the expression of miR-223. To determine whether antioxidants affect the expression of miR-223 in ADSCs induced by AGE-HSA, the cells were pretreated with antioxidants for 20 h and then treated with AGE-HSA (300 μg/ml) for 24 h. Expression of miR-223 was then determined by RT-PCR. As shown in Fig. 2B, antioxidant pretreatment significantly suppressed upregulation of miR-223 in ADSCs induced by AGE-HSA (P < 0.05).

### Role of miR-223 in the protective effects of antioxidants against AGE-HSA-induced apoptosis, caspase-3 activity, and oxidative stress levels in ADSCs

**Figure 3:**
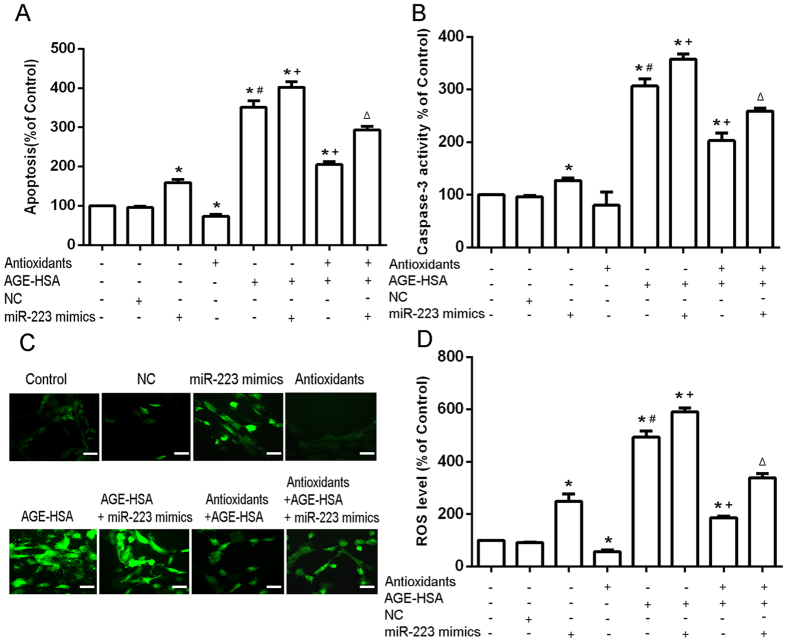
Role of overexpression of miR-223 in the protective effects of antioxidants on AGE-HSA-induced apoptosis of ADSCs. Cells were transfected with miR-223 mimics and/or pretreated with antioxidants and subsequently treated with AGE-HSA (300 μg/ml) for 24 h. The levels of apoptosis (**A**) and caspase-3 activity (**B**) were measured by ELISA. (**C**) Intracellular ROS generation was visualized under the fluorescence microscope. The scale bars represent 100 μm. (**D**) The level of DCF-sensitive ROS was measured by a flow cytometer. Each value is expressed as the mean ± SD of three independent experiments. *P < 0.05 vs. control (HSA 300 μg/ml), ^#^P < 0.05 vs. antioxidant pretreatment (3 mM NAC and 0.2 mM AAP), ^+^P < 0.05 vs. AGE-HSA (300 μg/ml), ^Δ^P < 0.05 vs. antioxidant pretreatment (3 mM NAC and 0.2 mM AAP) and AGE-HSA (300 μg/ml).

**Figure 4:**
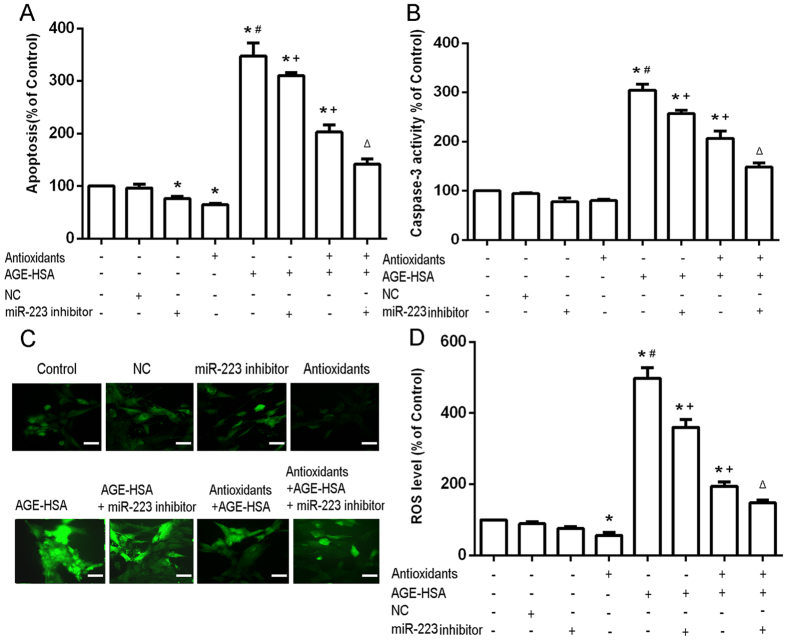
Role of downregulation of miR-223 in the protective effects of antioxidants on AGE-HSA-induced apoptosis of ADSCs. Cells were transfected with miR-223 inhibitors and/or pretreated with antioxidants and subsequently treated with AGE-HSA (300 μg/ml) for 24 h. The levels of apoptosis (**A**) and caspase-3 activity (**B**) were measured by ELISA. (**C**) Intracellular ROS generation was visualized under the fluorescence microscope. The scale bars represent 100 μm. (**D**) The level of DCF-sensitive ROS was measured by a flow cytometer. Each value is expressed as the mean ± SD of three independent experiments. *P < 0.05 vs. control (HSA 300 μg/ml), ^#^P < 0.05 vs. antioxidant pretreatment (3 mM NAC and 0.2 mM AAP), ^+^P < 0.05 vs. AGE-HSA (300 μg/ml), ^∆^P < 0.05 vs. antioxidant pretreatment (3 mM NAC and 0.2 mM AAP) and AGE-HSA (300 μg/ml).

To assess the role of miR-223 in response to AGE-HSA and antioxidant treatments, apoptosis and ROS generation were examined in ADSCs in the presence or absence of miR-223 mimics or inhibitors. MiR-223 expression was confirmed by RT-PCR. The results of apoptosis analysis showed that transfection of miR-223 mimics positively regulated the apoptosis induced by AGE-HSA (P < 0.05) (Fig. 3A). Moreover, the results of caspase-3 activity analysis showed that transfection of miR-223 mimics positively regulated the caspase 3 activity induced by AGE-HSA (P < 0.05) (Fig. 3B). Measurement of cellular ROS generation showed that transfection of miR-223 mimics positively regulated the ROS generation induced by AGE-HSA (P < 0.05) (Fig. 3C,D). In contrast, cells transfected with miR-223 inhibitors showed negative regulation of the apoptosis induced by AGE-HSA (P < 0.05) (Fig. 4A). Furthermore, transfection of miR-223 inhibitors negatively regulated the caspase 3 activity induced by AGE-HSA (P < 0.05) (Fig. 4B). Transfection of miR-223 inhibitors also resulted in negatively regulation of the ROS generation induced by AGE-HSA (P < 0.05) (Fig. 4C,D). To further examine whether miR-223 was required for the protective effects of antioxidants against apoptosis induced by AGE-HSA, cells were transfected with miR-223 mimics or inhibitors in the presence or absence of antioxidants and then treated with AGE-HSA (300 μg/ml) for 24 h. We found that the protection conferred by the antioxidants was diminished by upregulation of miR-223, but amplified by downregulation of miR-223 (Figs 3 and 4). These results indicated that miR-223 may act as a positive modulator of AGE-BSA-induced apoptosis in ADCSs, and that antioxidants inhibit AGE-HSA-induced apoptosis by downregulation of miR-223 in ADSCs.

### FGFR2 is a direct target of miR-223

**Figure 5:**
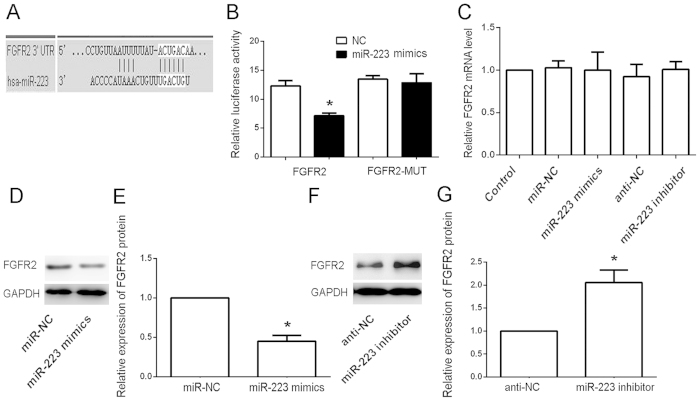
FGFR2 is a direct target of miR-223 in ADSCs. (**A**) miR-223 directly targeted FGFR2. (B) Analysis of the luciferase activities of FGFR2 and FGFR2-MUT in ADSCs transfected with miR-223 mimics or NCs. *P < 0.05.(**C**) mRNA expression analysis of FGFR2 in parental and transfected cells by RT-PCR. No significant difference in the levels of endogenous FGFR2 mRNA was found in transfected and parental cells normalized to an endogenous reference (GAPDH). (**D**,**E**) Protein expression analysis of FGFR2 in miR-223 mimic-transfected, NC-transfected, and parental cells by western blotting. A clear reduction in the level of endogenous FGFR2 protein was found in miR-223 mimic-transfected cells compared with NC-transfected cells normalized to an endogenous reference (GAPDH). (**F**,**G**) Protein expression analysis of FGFR2 in miR-223 inhibitor-transfected, NC-transfected, and parental cells by western blotting. A clear increase in the level of endogenous FGFR2 protein was found in miR-223 mimic-transfected cells compared with NC-transfected cells normalized to an endogenous reference (GAPDH).

Next, we identified the miR-223 target gene to gain a further insight into the molecular mechanisms of miR-223 in the protective effects of antioxidants against AGE-HSA-induced apoptosis. The public database-TargetScan (http://www.targetscan.org) was used to predict the potential target of miR-223. Because of a critically conserved binding site, *FGFR2* was selected for further examination (Fig. 5A). To confirm that FGFR2 is a direct target of miR-223, we constructed the luciferase reporter pGL3-FGFR2-3′-UTR. Scrambled target sites (pGL3-FGFR2-MUT) were used as controls for sequence specificity. The luciferase activity of the pGL3-FGFR2-30-UTR reporter was significantly suppressed in miR-223-transfected ADSCs compared with Negative Control (NC)-transfected cells normalized to a control vector containing Renilla luciferase, pRL-TK. In contrast, there was no significant difference in the relative luciferase activity of the pGL3-fibroblast-like growth factor receptor 2 (FGFR2)-MUT reporter in miR-223-transfected ADSCs compared with NC-transfected cells (P > 0.05) (Fig. 5B). These results showed that FGFR2 underwent direct negative regulation by miR-223 in ADSCs. In support of these results, we examined FGFR2 protein and mRNA levels in miR-223- or anti-miR-223-transfected cells, and their respective NC and parental cells by RT-PCR and western blotting, respectively. We observed a clear reduction in the level of endogenous FGFR2 protein in miR-223-transfected cells compared with NC-transfected and parental cells normalized to an endogenous reference, GAPDH (Fig. 5D). Overexpression of IGFR protein was also found in anti-miR-223-transfected cells compared with anti-NC-transfected and parental cells (Fig. 5F). These results demonstrated that miR-223 may target FGFR2 in ADSCs. Despite the effect of miR-223 on FGFR2 protein levels, no effect on FGFR2 mRNA levels was detected (Fig. 5C).

### FGFR2 is involved in the protective effect of antioxidants on AGE-HSA-induced apoptosis

**Figure 6:**
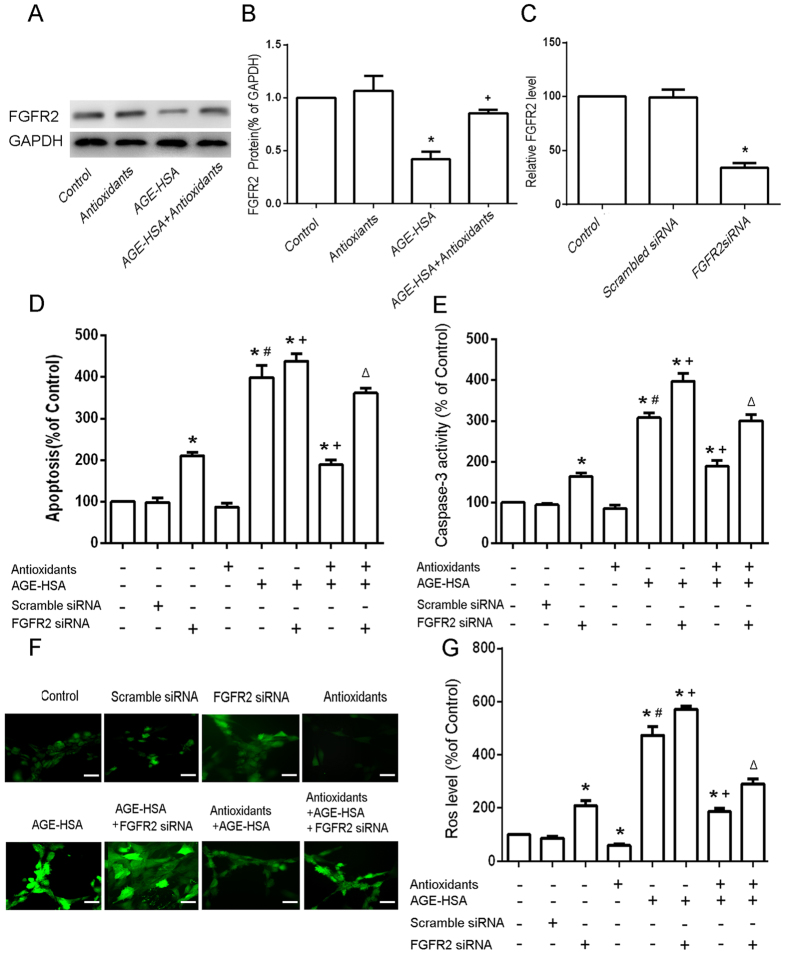
Effects of siFGFR2 on the protective effects of antioxidants against AGE-HSA-induced apoptosis in ADSCs. (**A**) Cells were pretreated with antioxidants followed by treatment with AGE-HSA (300 μg/ml) for 24 h. The protein expression level of FGFR2 was analyzed by western blotting. (**B**) Results from three independent western blots were quantified by ImageJ software. (**C**) FGFR2 silencing was verified by RT-PCR. FGFR2 siRNA-transfected ADSCs were pretreated with antioxidants followed by treatment with AGE-HSA (300 μg/ml) for 24 h. The levels of apoptosis (**D**) and caspase-3 activity (**E**) were measured by ELISA. (**F**) Intracellular ROS generation was visualized under the fluorescence microscope. The scale bars represent 100 μm. (**G**) The level of DCF-sensitive ROS was measured by a flow cytometer. Each value is expressed as the mean ± SD of three independent experiments. *P < 0.05 vs. control (HSA 300 μg/ml), ^#^P < 0.05 vs. antioxidants (3 mM NAC and 0.2 mM AAP), ^+^P < 0.05 vs. AGE-HSA (300 μg/ml), ^∆^P < 0.05 vs. antioxidants (3 mM NAC and 0.2 mM AAP) and AGE-HSA (300 μg/ml).

**Figure 7:**
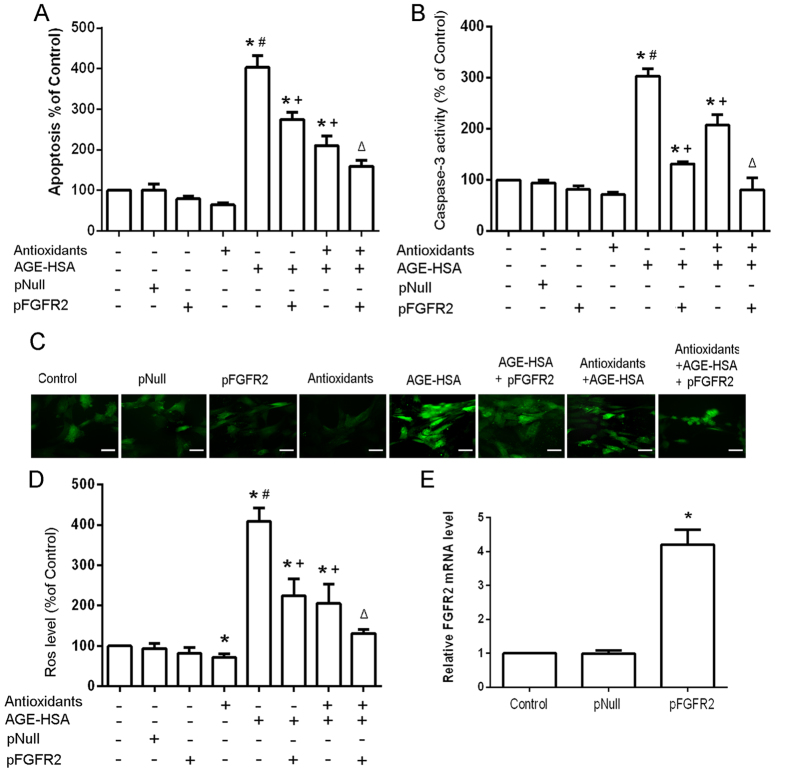
Effects of restoring FGFR2 on the protective effects of antioxidants against AGE-HSA-induced apoptosis in ADSCs. The levels of apoptosis (**A**) and caspase-3 activity (**B**) were measured by ELISA. (**C**) Intracellular ROS generation was visualized under the fluorescence microscope. The scale bars represent 100 μm. (**D**) The level of DCF-sensitive ROS was measured by a flow cytometer.(**E**) Fored expression of FGFR2 was verified by RT-PCR.PReceiver-E2F3 or an empty pReceiver vector-transfected ADSCs were pretreated with antioxidants followed by treatment with AGE-HSA (300 μg/ml) for 24 h. Each value is expressed as the mean ± SD of three independent experiments. *P < 0.05 vs. control (HSA 300 μg/ml), ^#^P < 0.05 vs. antioxidants (3 mM NAC and 0.2 mM AAP), ^+^P < 0.05 vs. AGE-HSA (300 μg/ml), ^Δ^P < 0.05 vs. antioxidants (3 mM NAC and 0.2 mM AAP) and AGE-HSA (300 μg/ml).

Next, we further examined whether the counteraction of miR-223 overexpression against the protective effect of antioxidants on AGE-HSA-induced apoptosis is mediated by FGFR2. The expression of FGFR2 in cells treated with 300 μg/ml AGE-HSA for 24 h after pretreatment with antioxidants for 20 h was analyzed by western blotting. In addition, the apoptosis and ROS production of FGFR2 knockdown ADSCs stimulated with AGE-HSA for 24 h after pretreatment with antioxidants for 20 h were assessed. FGFR2 expression in cells was decreased following AGE-HSA treatment for 24 h (P < 0.05) (Fig. 6A), and antioxidant pretreatment for 20 h rescored the expression of FGFR2 in ADSCs treated with AGE-HSA (Fig. 6B). Knockdown of FGFR2 clearly diminished the protective effect of antioxidants on AGE-HAS-induced apoptosis in ADSCs (P < 0.05)(Fig. 6D–G).The results revealed that overexpression of FGFR2 could argument the protective effect of antioxidants on AGE-HAS-induced apoptosis in ADSCs (Fig. 7). These data indicated that FGFR2 was involved in the protective effect of antioxidants on AGE-HSA-induced apoptosis in ADSCs.

## Discussion

Despite the fact that recent preclinical studies have shown beneficial effects of ADSC administration for treating a wide variety of diseases, including animal models of diabetes^5^,^21^,^22^,^23^, impairment of resident and recruited cell functions strongly contributes to delays in wound healing under diabetic conditions. A number of studies have suggested that diabetes mediates stem cell apoptosis and plays an important role in the pathogenesis of biophysical disorders^24^,^25^. However, the mechanism of diabetes in the impairment of ADSC functions and approaches has been not elucidated.

AGEs are noxious metabolic products that have been found to significantly accumulate in diabetic patients in comparison with normal individuals. They cause adverse effects on the growth of several cell types such as vascular endothelial cells and renal tubular epithelial cells. The elevated levels of AGEs in diabetic patients induce pathological changes such as promotion of EPC and endothelial cell apoptosis^26^. Our previous study confirmed that AGEs promote apoptosis of ADSCs *in vitro* via a receptor for the advanced glycation end-product (RAGE)-dependent p38 MAPK pathway^7^. The interaction of AGEs with RAGE regulates various cellular processes and leads to increased production of ROS. In this study, as expected, we found a significant increase in the production of ROS in AGE-HSA-treated cells compared with that in the control. Excessive production of ROS due to sustained oxidative stress in diabetic patients affects the survival of engrafted MSCs. The increase in ROS might damage proteins, DNA, and lipids of engrafted cells. In addition, increased ROS production induces superoxide in the cell membrane and damages cell membrane proteins, resulting in calcium influx and ultimately an abnormal cell structure, functions, and metabolic activity, as well as mitochondrial dysfunction or apoptosis^16^. Furthermore, as a second messenger, ROS stimulates important signal transduction pathways, regulates cell functions, and triggers signaling cascades, such as mitogen-activated protein kinase and NF-kB, which regulate gene expression and induce cell apoptosis^27^,^28^.

Co-treatment with NAC and AAP to prevent cell death and injury due to oxidative stress is largely based on *in vitro* observations. Turgeon *et al.* reported that probucol and antioxidant vitamins rescue ischemia-induced neovascularization in mice exposed to cigarette smoke by improving the functions of EPCs. Previous reports have also shown that addition of other antioxidants, such as epigallocatechin-3-gallate, curcumin, melatonin, and b-estradiol, reduce cellular oxidative stress and inhibit the apoptosis of MSCs^4^. In this study, we first identified the optimal combination of NAC and AAP (3 mM and 0.2 mM, respectively) to exert protective effects on ADSCs under AGE-HSA-induced oxidative stress. Based on these data, we hypothesized that antioxidants suppressed AGE-HSA-induced apoptosis in ADSCs. Under ROS production induced by treatment with 300 μg/l AGE-HSA for 24 h, we found significant decreases in the levels of apoptosis and caspase-3 activity when cells were pretreated with NAC and AAP for 20 h. These results are consistent with a report by Li *et al.* who found synergistic protection conferred by NAC and AAP against mitoptosis, necroptosis, and apoptosis in human MSCs treated with H_2_O_2_. Our data indicate that a reduction in oxidative stress levels by antioxidants might represent a novel therapeutic strategy to improve the survival of ADSCs exposed to AGEs.

Several recent reports have indicated the involvement of miRNAs in the regulation of intracellular ROS production and apoptosis signaling during diabetes^29^,^30^. Additionally, elevated expression of miR-21 mediates the oxidative stress response by increasing intracellular ROS levels and impairing nitric oxide bioavailability via targeting human superoxide dismutase-2 31. ROS production and apoptosis of AGE-treated endothelial cells involves suppression of miR-200b/miR-200c by upregulation of RhoA/ROCK2 signaling^32^. Apoptosis of human umbilical vein endothelial cells exposed to AGEs also involves miR-223 via targeting IGFR1^33^. Here, we aimed to clarify the biological role of miR-223 in ADSC apoptosis regulation by AGE-HSA and antioxidants. The results showed a significant increase in the miR-223 expression of ADSCs treated with AGE-HSA, but a reduction of in miR-223 expression when cells were pretreated with antioxidants before treatment with AGE-HSA. Reintroduction of miR-223 by transient transfection or siRNA silencing of the target gene (FGFR2) blocked the protective effects of antioxidants on AGE-HSA-induced apoptosis and increased the production of ROS, while increasing the level of apoptosis and activity of caspase-3 in ADSCs. Our results support the notion that protective effects of antioxidants on AGE-HSA-induced apoptosis in ADSCs are partially mediated by a reduction in miR-223 expression.

In conclusion, the present study suggests that miR-223 transfection bypasses antioxidant suppression of AGE-induced apoptosis at least partially through upregulation of FGFR2 expression. Downregulation of miR-223, through its positive effects on ADSCs, might help to restore the ability to heal wounds in diabetic individuals. Whether similar effects can be obtained in other clinical situations involving increased oxidative stress levels such as atherosclerotic diseases and hypercholesterolemia remains to be determined. If this is the case, modification of oxidative stress levels by miRNAs might represent a novel therapeutic strategy to restore impaired cell functions and promote wound healing by ADSCs exposed to AGEs.

## Materials and Methods

### Cell culture and treatments

All the methods were carried out in accordance with the approved guidelines and all experimental protocols were approved by the ethics committee of China Medical University. Adipose tissue samples were obtained with informed consent from 10 patients at the Shengjing Hospital of China Medical University. Cells were isolated and harvested as described previously^7^. ADSC identities were confirmed by cell surface markers. For NAC and AAP (both purchased from Sigma Co., MO, USA) co-treatment, ADSCs were pretreated with 3 mM NAC and 0.2 mM AAP for 20 h, followed by incubation in medium containing 300 μg/ml AGE-HSA (Sigma) for 24 h.

### Transfection of miRNA mimics and inhibitors, siRNAs, and pReceiver FGFR2

Cells (5 × 10^5^)were seeded in a 6-well plate (Nest Biotechnology, Hong Kong, China). At 70% confluence, the cells were transfected with miR-223 mimics, miR-223 inhibitors, or a negative control. After 20 h, the cells were treated with or without 300 μg/ml AGE-HSA. Transfection of miR-223 mimics or inhibitors was carried out as described previously^11^. Analyses were performed at 24 h after transfection. siRNAs targeting fibroblast growth factor receptor 2 (FGFR2) and a control (scramble siRNA) were purchased from Santa Cruz Biotechnology. Transfection of 50 nM siRNA or scramble siRNA was performed as described previously. The human FGFR2-coding sequence excluding the 3′-UTR was inserted into a pReceiver vector (Genecopoeia, USA) to construct the pReceiver-FGFR2 vector. The cells were transfected with pReceiver FGFR2 or an empty pReceiver vector. All results are representative of three independent experiments.

### Apoptosis assay

For apoptosis assays, cells were seeded in 96-well plates at a density of 2 × 10^4^ cells per well. At approximately 80% confluence, the cells were cultured in high glucose DMEM medium (GIBCO, Gaithersburg, MD, USA) supplemented with 10% fetal bovine serum (FBS, GIBCO, Gaithersburg, MD, USA). To induce apoptosis, ADSCs were exposed to AGE-HSA (300 μg/ml) for 24 h. To determine the role of antioxidants in apoptosis, ADSCs were pretreated with 3 mM NAC and 0.2 mM AAP for 20 h, and then apoptosis was induced by treatment with 300 μg/ml AGE-HSA for 24 h. The level of apoptosis was determined using the Cell Death Detection ELISAPLUS kit (Roche Applied Science, Indianapolis, IN, USA), which detects cytoplasmic histone-associated DNA fragments, according to the manufacturer’s instructions.

### Caspase-3 activity assay

As a marker of apoptosis, caspase-3 activation was assessed using the Caspase-Glo3/7 Assay (Promega, Madison, USA) according to the manufacturer’s instructions.

### Measurement of intracellular ROS

Intracellular ROS and O_2_ generation were assessed using 2′,7′-dichlorofluorescein diacetate (DCFH-DA, Sigma) as described previously^34^. Briefly, ADSCs grown in 10-cm plates were subjected to the various culture conditions described above. The medium was replaced with control medium containing 10 μM DCFH-DA, and the cells were incubated for 30 min in the dark. Intracellular ROS generation was then visualized under a fluorescence microscope (Olympus, Tokyo, Japan). DCF fluorescence was measured by a flow cytometer. Data were normalized to the control values.

### RNA extraction and reverse transcription-polymerase chain reaction (RT-PCR)

RT-PCR was performed as described previously^35^. Briefly, total RNA was extracted from each sample using Trizol reagent (Invitrogen, CA, USA) according to the manufacturer’s instructions. The quality of the isolated RNA was checked by agarose gel electrophoresis. RNA concentrations were determined by measuring the optical density at 260 and 280 nm. To analyze miR-223 expression, RT- PCR was performed using specific stem-loop RT primers from a Hairpin-in miRNA qPCR Quantitation Kit (GenePharma, Shanghai, China). Quantitative real-time PCR was performed using the same kit on the Applied Biosystems 7500 system. U6 was used as an internal control. To analyze FGFR2 mRNA expression, cDNA synthesis was carried out using random hexamer primers and MMLV reverse transcriptase under the conditions recommended by the manufacturer (Invitrogen). Specific primers were designed using Gene Runner software (Hastings Software, Inc.). Glyceraldehyde-3-phosphate dehydrogenase (GAPDH) was used as an internal control. Fold changes of both miRNA and mRNA expression were calculated using the 2^−ΔΔCt^ method. Primer sequences and PCR conditions are shown in Table 1.

### Dual luciferase reporter assay

A dual luciferase reporter assay was performed as reported previously^36^. Briefly, cells were seeded in 96-well plates and co-transfected with the pMir-Report luciferase vector, pRL-TK Renilla luciferase vector, and miR-223 mimics as reported previously. After 48 h, the luciferase activities were determined with the Dual Luciferase Reporter Assay System (Promega). All results are representative of three independent experiments.

### Western blot analysis

Proteins were isolated from harvested cells by mechanical disruption and the Mammalian Cell Lysis Kit (Sigma) according to the manufacturer’s instruction. The proteins were separated on 1-mm NuPage Novex 10% Bis-Tris gels using the NuPage MOPS SDS Buffer Kit (Life Technologies, Carlsbad, CA, USA) followed by electrotransfer to 0.2-mm nitrocellulose membranes (Pall, Port Washington, WI, USA). Nonspecific binding sites were blocked with 5% bovine serum albumin in PBS for 1 h at room temperature. The membranes were then incubated with diluted FGFR2 (1:1000; Cell Signaling Technology, Inc.) at 4 °C overnight. After three washes with PBS containing 0.5% Tween-20, the membranes were incubated with a diluted horseradish peroxidase-conjugated secondary anti-rabbit (GE Healthcare, Buckinghamshire, UK) at room temperature for 2 h. The signal was visualized with enhanced chemiluminescent reagent (Amersham Biosciences, Piscataway, NJ, USA). As a protein loading control, blots were stripped and stained for GAPDH using an anti-GAPDH antibody (1:2000, AbCam, Cambridge, MA, USA).

### Statistical Analysis

Statistical analysis was performed using SPSS version 19.0 software. Data are presented as the means ± standard deviation (SD). Univariate comparisons of the means were performed using the Student’s t-test with a significance threshold of P < 0.05. The data shown in figures are representative of three independent experiments.

## Additional Information

**How to cite this article**: Wang, Z. *et al.* Antioxidants inhibit advanced glycosylation end-product-induced apoptosis by downregulation of miR-223 in human adipose tissue-derived stem cells. *Sci. Rep.* **6**, 23021; doi: 10.1038/srep23021 (2016).

## Figures and Tables

**Table 1:** Sequences of primers and PCR conditions.

	Primer sequence	Product size (bp)	Ta (°C)/cycles
FGFR2	S: 5′-GGTGGCTGAAAAACGGGAAG-3′	104	60.5/38
	AS: 5′-AGATGGGACCACACTTTCCATA-3′		
GAPDH	S: 5′-ACCACAGTCCATGCCATCAC-3′	452	56.0/26
	AS: 5′-TCCACCACCCTGTTGCTGTA-3′		

S, sense; AS, antisense; Ta, annealing temperature.
